# Editorial: Mitochondrial damage interacts with the immune system and tumor microenvironment

**DOI:** 10.3389/fimmu.2025.1646413

**Published:** 2025-06-30

**Authors:** Xundong Lin, Xin Yuan, Yiheng Luo, Haihong Fang, Weihong Guo

**Affiliations:** Nanfang Hospital, Southern Medical University, Guangzhou, China

**Keywords:** cancer, mitochondrial damage, immune response, tumor microenvironment, cell death, immune therapy

Despite the clinical success of immunotherapy in certain cancers, patient sensitivity and resistance remain persistent challenges. As the primary energy powerhouses of eukaryotic cells, mitochondria exhibit dysfunction and metabolic perturbations that alter endogenous molecular expression (1, 2). Mitochondria also extensively participate in emerging cell death processes such as ferroptosis and cuproptosis, consequently reshaping the infiltration and functional state of tumor immune cells. Exploring mitochondria-associated targets and their crosstalk with cell death pathways represents a potential approach to overcome immunotherapy resistance.

This Research Topic presents a collection of innovative mitochondria-centered studies and reviews oriented toward cancer immunotherapy. We aim to enhance scholarly understanding of mitochondrial impacts within tumor micro environments(TME) and advance the development of cancer immunotherapy.

Tumors manifest three immune phenotypes defined by immune cell infiltration and functionality: immune-inflamed, immune-excluded, and immune-desert. Paradoxically, immune-inflamed tumors exhibit elevated expression of PD-L1, TILs, B cells, and intact antigen presentation machinery compared to the other two phenotypes, correlating with superior responses to cancer immunotherapy (3). Therapeutically targeting specific genes in tumor cells to reprogram the immunosuppressive microenvironment represents a promising strategy. Chen et al. established a seven-gene risk scoring model to stratify lung adenocarcinoma (LUAD) patients into high- and low-risk cohorts, thereby investigating the impact of N6-methyladenosine (m^6^A) modification on the TME. Their data showed that knockout of METTL3, a core component of RNA m^6^A modification, increased PD-L1 expression and enhanced T-cell cytotoxicity. Simultaneously, they observed that METTL3-mediated m^6^A modifications regulate candidate genes. These dual effects collectively strengthen the rationale for targeting METTL3 in LUAD therapy. The model additionally demonstrated that high-risk tumors manifested an immune-inflamed phenotype characterized by elevated TMB and immune cell infiltration, yet concurrently exhibited a suppressive immune state marked by downregulated HLA-D family expression. This profile suggests increased susceptibility to ICIs in high-risk patients, supporting combinatorial chemotherapy-immunotherapy strategies to circumvent resistance. Collectively, This model establishes a framework for the precision selection of immunotherapy candidates and the advancement of personalized therapeutic strategies.

Accumulating evidence indicates that mitochondrial-targeted therapy represents a viable strategy to improve cancer immunotherapy (4–6). Ma et al. identified the mitochondria-related gene ISCU as a candidate target for modulating the immune microenvironment and predicting diagnosis/prognosis in cervical cancer. ISCU potentially regulates immune cells within the TME by mediating iron metabolism to modulate ROS. Using a risk scoring model centered on mitochondria-metabolism genes (with ISCU as the core), the authors found that high-risk patients exhibited poorer prognosis due to an immunosuppressive microenvironment enriched with Treg cells. Conversely, low-risk patients showed favorable prognosis attributed to enhanced anti-tumor immunity from increased macrophage infiltration. Therapeutically, high-risk patients demonstrated greater sensitivity to immunotherapy, potentially owing to elevated TMB. Low-risk patients exhibited heightened sensitivity to targeted agents.

Mitochondria extensively participate in diverse cell death modalities. Wang et al. discovered that mitophagy-related genes COASY, FTSJ1, and MOGS potentially influence the TME through reciprocal regulation with CD8^+^ T cells and B cells. Concurrently, these genes were found to be closely associated with FOXM1/RB pathways governing cancer cell cycle and proliferation. Notably, these three genes are expressed in various cancer cells and hold broad application prospects, but their specific molecular mechanisms and clinical validation still require further experimental studies and validation. Zeng et al. elucidated the bidirectional regulatory role between mitochondria and TME in their published review. Mitophagy elevates inner mitochondrial membrane permeability, facilitating the release of apoptotic proteins that induce apoptosis. Conversely, hypoxia within the TME promotes Drp1-mediated mitochondrial damage, which exacerbates hypoxic conditions to establish a self-perpetuating cycle. Mitochondrial damage-generated ROS additionally drive ferroptosis. The review summarized the complex effects of mitochondria on ferroptosis. For example, PINK 1/Park 2 and BNIP 3-mediated mitophagy inhibit ferroptosis, while the mitochondrial glutaminolysis-TCA-ETC axis is a key pathway that promotes ferroptosis. Beyond ferroptosis, emerging mitochondria-linked death modalities including cuproptosis and pyroptosis regulate the TME (7, 8).

Shen et al. outlined the independent and interactive effects of mitochondria, lysosomes, and endoplasmic reticulum on proliferation, invasion, and therapy resistance in gynecologic malignancies. This evidence advances a comprehensive understanding of mitochondrial mechanisms within complex TME networks and supports the development of organelle-targeted therapies. Xing et al. employed a comprehensive analysis of 2039 publications to demonstrate how mitochondrial damage remodels the immunosuppressive tumor microenvironment by regulating ROS generation, cell cycle arrest, and immune cell function. Their work mechanistically reveals mitochondria’s pivotal role and proposes strategies like photodynamic therapy and mitochondria-targeted nanodrugs to enhance immunotherapy response, thereby providing novel targets and directions to overcome current immunotherapy resistance bottlenecks.

In summary, This Research Topic highlights the complex role of mitochondria in regulating the TME and immune response. Mitochondrial damage, metabolic alterations, interactions with other organelles, or engagement in novel cell death pathways may each contribute to reprogramming the immunosuppressive tumor microenvironment. This lays the theoretical foundation for predicting the efficacy and prognosis of immunotherapy in patients and developing mitochondria-directed cancer immunotherapy strategies.
